# The International Congress from an English Standpoint

**Published:** 1890-10

**Authors:** 


### The International Congress from an English Standpoint.

In a leading editorial in the *British Medical Journal* of August 16, there occurs the following reference to the recent Congress at Berlin:

"*Congressus haud impar*—Berlin has had to sustain the stress of a gathering of medical men of all nations, in numbers hitherto unprecedented. They invaded the metropolis of the German Empire in hosts from every part of the world. They came, they saw, and they admired. Nothing could have exceeded the overflowing hospitality, the generous and cordial welcome, the thoughtful courtesy, and the minutely elaborate preparations with which the social and scientific needs of the invading hosts were met. All were alike made to feel this welcome; so that, whether for its remarkable assemblage of the most eminent representatives of cosmopolitan medical science, or for its endless round of scientific and social activity, the Berlin Congress must be pronounced to have far exceeded any which has preceded it; and it will certainly be difficult in the future for any capital to surpass the feat which Berlin has accomplished."

